# A combined experimental and numerical study on upper airway dosimetry of inhaled nanoparticles from an electrical discharge machine shop

**DOI:** 10.1186/s12989-017-0203-7

**Published:** 2017-07-12

**Authors:** Lin Tian, Yidan Shang, Rui Chen, Ru Bai, Chunying Chen, Kiao Inthavong, Jiyuan Tu

**Affiliations:** 1 School of Engineering – Mechanical and Automotive, RMIT University, Bundoora, VIC Australia; 2 CAS Key Lab for Biomedical Effects of Nanomaterials and Nanosafety & CAS Center for Excellence in Nanoscience, Beijing Key Laboratory of Ambient Particles Health Effects and Prevention Techniques, National Center for Nanoscience and Technology of China, Beijing, China; 3 School of Architecture, Tsinghua University, Beijing, China

**Keywords:** Inhalation toxicity, Nanoparticles, Human upper airways, Particle dosimetry, Particle size distribution, Computational fluid dynamics (CFD)

## Abstract

**Backgrounds:**

Exposure to nanoparticles in the workplace is a health concern to occupational workers with increased risk of developing respiratory, cardiovascular, and neurological disorders. Based on animal inhalation study and human lung tumor risk extrapolation, current authoritative recommendations on exposure limits are either on total mass or number concentrations. Effects of particle size distribution and the implication to regional airway dosages are not elaborated.

**Methods:**

Real time production of particle concentration and size distribution in the range from 5.52 to 98.2 nm were recorded in a wire-cut electrical discharge machine shop (WEDM) during a typical working day. Under the realistic exposure condition, human inhalation simulations were performed in a physiologically realistic nasal and upper airway replica. The combined experimental and numerical study is the first to establish a realistic exposure condition, and under which, detailed dose metric studies can be performed. In addition to mass concentration guided exposure limit, inhalation risks to nano-pollutant were reexamined accounting for the actual particle size distribution and deposition statistics. Detailed dosimetries of the inhaled nano-pollutants in human nasal and upper airways with respect to particle number, mass and surface area were discussed, and empirical equations were developed.

**Results:**

An astonishing enhancement of human airway dosages were detected by current combined experimental and numerical study in the WEDM machine shop. Up to 33 folds in mass, 27 folds in surface area and 8 folds in number dosages were detected during working hours in comparison to the background dosimetry measured at midnight. The real time particle concentration measurement showed substantial emission of nano-pollutants by WEDM machining activity, and the combined experimental and numerical study provided extraordinary details on human inhalation dosimetry. It was found out that human inhalation dosimetry was extremely sensitive to real time particle concentration and size distribution. Averaged particle concentration over 24-h period will inevitably misrepresent the sensible information critical for realistic inhalation risk assessment.

**Conclusions:**

Particle size distribution carries very important information in determining human airway dosimetry. A pure number or mass concentration recommendation on the exposure limit at workplace is insufficient. A particle size distribution, together with the deposition equations, is critical to recognize the actual exposure risks. In addition, human airway dosimetry in number, mass and surface area varies significantly. A complete inhalation risk assessment requires the knowledge of toxicity mechanisms in response to each individual metric. Further improvements in these areas are needed.

## Background

Exposure to nanoparticles in the workplace is a health concern to occupational workers where there is an increased risk of developing respiratory, cardiovascular, and neurological disorders [1]. Confirmed inhalation hazards include the notorious asbestos, with low dosage, causing severe health consequences [2]. The onset of “manganism”, a clinical diagnosed neuro-toxin caused by high level exposure to manganese containing particles, were reported in occupational workers conducting mining, ore grinding and smelting activities [3, 4]. In addition to confirmed cases, there have been discussions on the link between sub-clinical human functional impairment and chronic low dose metal particle exposures [5–7]. Similar concerns were also reported in the office environment where the increased usage of modern electrophotography machines elevates the health risks of office workers on inhalation exposure to the emitted nanoparticles during xerographic processes [8, 9]. Electrical discharge machining (EDM) is one of the most important manufacturing processes in the die and mold industry for delicate concave shapes which traditional machining cannot achieve [10]. Rather than using mechanical forces, EDM utilizes high voltage between the “wire” electrode and the conductive metal piece to cause high energy sparks which remove the material by melting and erosion. This high energy electrophysical process is more likely to generate pollutant by-product in nanoscale [11].

Based on animal inhalation study and human lung tumor risk extrapolation, National Institute for Occupational Safety and Health (NIOSH, USA) [12] recommended exposure limits to fine (diameter > 0.1 μm) and ultrafine (diameter ≤ 0.1 μm) titanium dioxide particles as 2.4 mg/m^3^ and 0.3 mg/m^3^ in normal working conditions. German Institute for Occupational Safety and Health of the German Social Accident Insurance (IFA) [13] established the benchmark limits for ultrafine particle concentrations in workplaces based on state of the art knowledge of measurement and exposure risks. It states that, for ultrafine (1 to 100 nm) metal, metal oxide and other biopersistent granular nanomaterials with a density > 6000 kg/m^3^, a particle number concentration of 20,000 particles/cm^3^ should not be exceeded. For ultrafine particles with a density below 6000 kg/m^3^, a particle number concentration below 40,000 particles/cm^3^ should be imposed. Other recommendations include 10 mg/m^3^ by American Conference of Governmental Industrial Hygienists (ACGIH) [14] and 15 mg/m^3^ by the Occupational Safety and Health Administration (OSHA, USA) [15] for total inhalable particles (diameter ≤ 100 μm). For respirable particles that can penetrate to the alveolar region (diameter ≤ 10 μm), ACGIH and OSHA refine the exposure limit to 3 and 5 mg/m^3^ respectively [12]. In summary, current exposure standards are focused on ultrafine nanoparticles of 1 to 100 nm, and the recommendations are either on the total mass or number concentrations. Effects of the particle size distribution and the implication to regional airway dosages are not elaborated.

In addition to animal and experimental studies, computational fluid dynamics (CFD) are frequently used for investigating detailed human inhalation and particulate transport processes. Compared to experiments, computer simulations are significantly less restrictive from time, cost and ethical perspectives. It allows decomposition of the complex physical phenomenon into focused areas where details of the particle-pulmonary interactions can be derived and integrated. Respiratory anatomy, airflow and particle transport and deposition are the main focused areas where a broad range of CFD studies were reported in past 2 decades. Heistracher and Hofmann (1995) proposed a physiologically realistic human bronchial airway bifurcation model [16]. Tian and Ahmadi (2012) extended the model for multi-level bronchial bifurcations where entire lung can be constructed sequentially [17]. For nasal airways, Zamankhan et al. (2006) and Inthavong et al. (2009) presented methodology of reconstructing human nasal cavities from casts and CT scans respectively [18, 19]. Detailed flow patterns and particle transport characteristics around the human body can be found in the work of Kennedy and Hinds (2002), Anthony and Flynn (2006), Se et al. (2010), Inthavong et al. (2012, 2013), and Ghalati et al. (2012) [20–25]. Inside the human respiratory system, Katz and Martonen (1996), Zhang and Kleinstreuer (2001), Hofmann et al. (2003), Tian and Ahmadi (2012, 2013), and Inthavong et al. (2010) [17, 26–29] employed computational models to investigate the airflow, and particle transport and deposition in human tracheobronchial airways. Subramaniam et al. (1998), Matida et al. (2003), Zamankhan et al. (2006), Xi and Longest (2008), Inthavong et al. (2011), Ge et al. (2012) and Tian et al. (2016) [18, 30–35] applied CFD methods in the human nasal/head airways for airflow and particle transport analysis. To evaluate the influence of breathing pattern on particle deposition, Häuβermann et al. (2002) and Inthavong et al. (2010) performed particle transport modeling in nasal and tracheobronchial airways respectively [36, 37]. CFD also plays important role in the study of non-spherical particle transport behavior in human airways. Tian et al. (2013, 2016), Inthavong et al. (2008), and Dastan et al. (2013) were among the few who investigated fibrous and agglomerated particle deposition in human nasal and tracheobronchial airways using CFD-DPM method [29, 34, 53, 38–40]. Recently, CFD-DEM has gained growing interest in studying non-spherical particle dynamics [41]. It has the potential to be applied in human inhalation study. These computational investigations provide detailed descriptions of flow and particle features and allow wider coverage of flow and particle conditions, which would otherwise be difficult to infer from experimental measurements.

While most of the computational analysis provides valuable information on detailed flow pattern, particle trajectories and deposition statistics, there has been no study incorporating a realistic inhalation profile accounting for exposure to particle size distribution to inhaled dosimetry. In this research, a combined experimental and numerical study in the upper airway dosimetry of ultrafine particles in an electrical discharge machine shop was performed. The real time evolution of particle concentration and size distribution in the range from 5.52 to 98.2 nm during normal operation of electrical discharge machining was measured during a typical working day. Under these conditions, human inhalation simulations were performed in a physiologically realistic nasal and upper airway replica. Respiratory health risk was determined by regional dosimtries in context of exposure limits recommended by NIOSH, ACGIH, OSHA and IFA. In addition, dose metric relationships with respect to particle number, mass and surface area were analyzed. Based on the simulation data, empirical equations were developed to predict the local dosimetry of the inhaled nanoparticles in human nasal, laryngeal and deeper airways. The combined experimental and numerical study is the first to establish a realistic exposure condition, and under which, detailed dose metric studies were performed. The developed empirical equations will be useful for future nanoparticle dose-deposition prediction in inhalation risk assessments.

## Methods

### Particle measurement in an electrical discharge machine shop

**Fig. 1:**
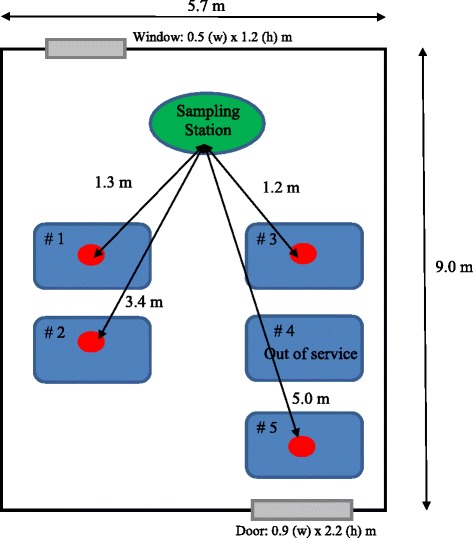
Floor plan of the WEDM machine shop (dimensions not to scale)

Located in Beijing, China, the 3.8 m high machine shop hosts five wire-cut electrical discharge machineries (WEDM) manufacturing hardened metal pieces of desired shape during regular working hours (Fig. 1) (WEDM#1-Beijing AgieCharmills Industrial Electronics FW2; WEDM#2 and #3 – Shanghai Troop Group Photoelectric Technology TP-25ZT and TP3271; WEDM#5-Beijing Ninva NH7120ND; WEDM#4 – out of service). The high voltage between the “wire” electrode and the conductive metal piece causes high energy sparks which remove the material by melting and eroding processes. A dielectric liquid (DIC-206, Beijing Hua Ye Oil Limited, China) was used to flush out particle debris as well as restore the electrode potentials. Particle measurements took place during normal working hours from 8:00 to 17:30 in winter. Due to the cold weather condition, window in the machine shop was closed during the working hours, however left open overnight. The door was normally closed with occasional opening by the single machine operator during breaks, including a regular lunch break from 12:00 to 13:30. There was no mechanical ventilation in the workshop and no personal protective equipment was used by the operator due to minimal visible fume emission. The sampling station was located in the breathing zone about 1.5 m high and 1.2 to 5.0 m away from the electrical discharge machines.

The sampling station hosts a suite of aerosol instruments, and the ultrafine particle concentration was measured by a Scanning Mobility Particle Sizer (SMPS, TSI Model 3936, USA), consisting of a Water-Based Condensation Particle Counter (CPC, TSI Model 3788, USA), an Electrostatic Classifier (EC, TSI Model 3080, USA), a Nano Differential Mobility Analyzers (DMA, TSI Model 3085, USA) and a long DMA (TSI Model 3081, USA). Both the Nano and long DMAs detect particles up to 10^8^ particles/cm^3^ in real time, with a range from 2 to 100 nm, and 14 to 675 nm respectively. Larger particles >1 μm were eliminated by a pre-conditioner impactor at a setting of 0.0457 cm. The DMA Sheath Flow was 7 L/min. Before each field measurement, “zero” calibration was conducted by using a high efficiency particular air filter (HEPA). A diffusion loss correction was applied to account for the nanoparticle losses in the sample lines based on previously described method [42]. In addition to the particle concentration instrumentation, a Micro-Orifice Uniform-Deposit Step Size Impactors (MOUDI, Model 125B NanoMoudi-II, MSP Corporation, USA) was used to collect aerosol particle samples for morphology analysis. The airborne particles were captured on the polycarbonate filters (Φ 47 mm, 0.22 μ, Munktell Inc., Sweden), 2 Scanning Electron Microscopic (SEM) wafers (4 mm × 4 mm, Zhongxing Bairui Inc., China), and 2 Transmission electron microscopy (TEM) grids (200-mesh molybdenum with carbon film, Zhongxing Bairui Inc., China) at a steady flow rate of 10 L/min by using a vacuum pump (Sogevac SV10-16 B, Leybold Vacuum GmbH Co., Ltd., Germany). Offline examination of the wafer was performed with SEM (S-4800 N, HITACHI Inc., Japan). Further detail of the WEDM measurement was given in [43].

### Human nasal and upper respiratory airway Modeling

**Fig. 2:**
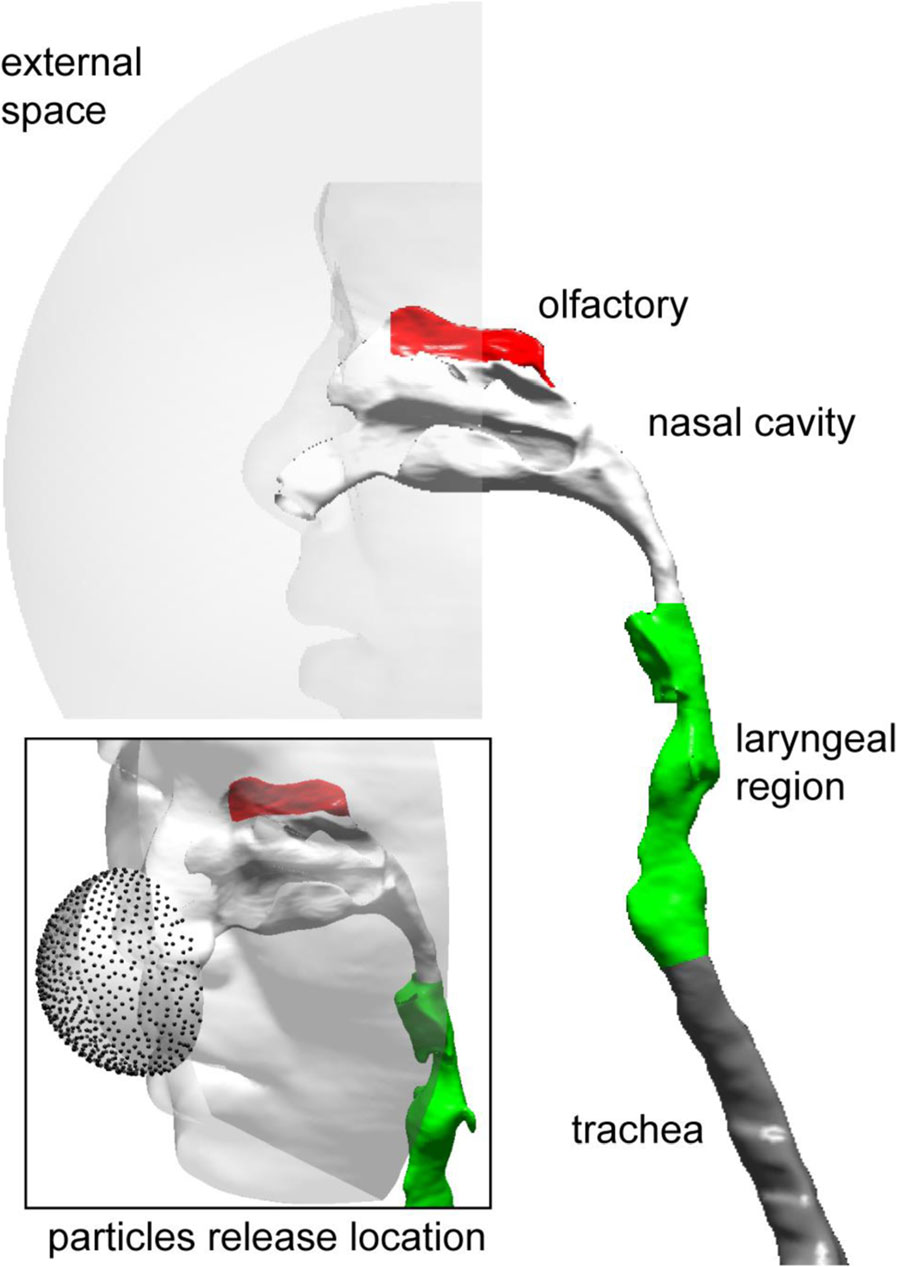
Human nasal and upper respiratory airway model

A CFD model of the upper respiratory airway containing facial features, the nasal cavity, larynx, trachea and first bifurcation of the bronchial airway tree was developed from CT scans [36, 44, 45] (Fig. 2). Each model of the respiratory airway was connected to form a contiguous path via nostrils, from the external space to the end of the larynx region. The larynx region was extended to the first lung bifurcation to allow sufficient flow recovery and improve numerical convergence in the CFD solution. The respiratory airway was added to a realistic human face exposed to the external surroundings containing airborne particles from the electrical machine shop. Shang et al. (2015) [45] showed that the airflow has negligible influence on particle trajectory outside the breathing zone. In this study, particles were uniformly released on a hemisphere (of radius 3 cm) with the center at the nose tip, resembling the release condition of Doorly et al. (2008) [46]. A high quality mesh (minimum orthogonality >0.1) incorporating prism layers was applied to the bounding respiratory walls, and tetrahedral unstructured mesh filled the airway passage. The final model is shown in Fig. 2, which consists of 7 million cells. Further detail of the computational model was given in [22].

### Fluid flow simulation

The current study employed a steady inhalation model with the assumption that particle deposition mainly occurs during the inhalation phase [36]. It is worth to note that breathing pattern was shown to affect deposition for micron range particles between 1 and 5 μm [37], however, the effect toward nanoparticle deposition was still not fully understood and requires further investigation. Mild cardiac load was assumed as the machine operator was mainly standing with occasional walking to attend the metal pieces. Laminar flow condition was considered and inspiration flow rates from 3 to 15 L/min were included. The wide coverage of the breathing rates is to facilitate the development of the empirical equations.

The airflow was simulated using Ansys-Fluent v16.2. The surrounding walls were set to atmospheric pressure and inhalation was initiated by a negative pressure difference at the bronchial bifurcation outlet. This allowed the ambient flow field to be influenced only by the inhaled air. The continuity and momentum equation of the fluid flow are:

1 $$ \frac{\partial }{\partial {x}_i}\left(\rho {u}_i\right)=0, $$

2 $$ \rho\;{u}_j\frac{\partial {u}_i}{\partial {x}_j}=-\frac{\partial p}{\partial {x}_i}+\frac{\partial }{\partial {x}_j}\left[\mu \frac{\partial {u}_i}{\partial {x}_j}\right]. $$

where *ρ*, *u* and *p* are density, velocity and pressure of the air, respectively. A second order upwind scheme was used to approximate the momentum equation, while the pressure–velocity coupling was handled through the SIMPLE method. Further detail of the fluid flow modeling was given in [47].

### Particle simulation

The Lagrangian particle tracking method is used where each particle’s trajectory is computed. The particle equation is:

3 $$ \frac{d{\boldsymbol{u}}_{\boldsymbol{p}}}{ d t}=\frac{1}{C_c}{\boldsymbol{\mathsf{F}}}_{\boldsymbol{\mathsf{D}}}+\frac{\boldsymbol{\mathsf{g}}\left({\rho}_p-\rho \right)}{\rho_p}+{\boldsymbol{\mathsf{F}}}_{\boldsymbol{\mathsf{L}}}+{\boldsymbol{\mathsf{F}}}_{\boldsymbol{\mathsf{B}}} $$

here ***u***_***p***_ is the particle velocity, *t* is the time, ***g*** is the gravitational constant, *ρ*_*p*_ is the particle density. In this study, both gravitational and buoyancy forces can be neglected. ***F***_***D***_ is the drag force given by *18 μ(* ***u***_***p***_ ***u*** *)/(d* ^*2*^ρ_*p*_ *),* here *d* is the particle diameter. *C*_*c*_ in Eq. (3) is the Cunningham correction given by:

4 $$ {C}_c=1+\frac{2\lambda}{d}\left(1.257+0.4{e}^{\left(-1.1 d/2\lambda \right)}\right), $$

λ is the molecular mean free path. ***F***_***L***_ in Eq. (3) is the Saffman lift force, and ***F***_***B***_ is the Brownian diffusion force with amplitude of $\zeta \sqrt{\pi {S}_0/\Delta t}$, here *ς* is a zero mean, unit variance independent Gaussian random numbers. ∆t is the time-step for particle integration and *S*_*o*_ is a spectral intensity function [48]:

:

5 $$ {S}_o=\frac{216\nu kT}{\pi^2\rho\;{d}^5{\left(\frac{\rho_p}{\rho}\right)}^2{C}_c}. $$

*ν* is the fluid kinematic viscosity, *k* is the Boltzmann constant, and *T* is the absolute temperature of the inspiratory air in the nasal cavity. The simulation was carried out with Ansys-Fluent v16.2 discrete phase model (DPM).

With the closed window, door and the lack of mechanical ventilation during the machining process, a homogeneous dispersion of the airborne particles was assumed in the breathing zone. For this study, statistically independent 100,000 uniform concentrated mono-dispersed airborne particles for each particle size, in a hemispheric profile (Fig. 2), were released. Particles of 1, 1.5, 2, 3, 5, 10, 15, 20, 30, 40, 50, 70 and 100 nm were included in the study. All particles entered the human nasal airway. Deposition onto the respiratory walls occurred when the particle was within *d/2* distance away from the surface. Here *d* was the particle diameter.

### Model validation

The particle Eq. (3) was solved by stepwise integration over discrete time steps yielding a new particle velocity at each time step. Inthavong et al. (2016) [49] identified the sensitivity of nanoparticle diffusion behavior in Lagrangian tracking to the integral time step factor, mesh size and flow condition. A methodology of selecting the most appropriate time step factors to achieve optimal Lagrangian tracking outcome was proposed and verified in a pipe and a human pharynx model [49].

In Ansys-Fluent, the length scale factor of integration, *L*_*s*_ controls the integration time step size and Δt is a function of the particle velocity and the continuous airflow phase velocity:

6 $$ \Delta t=\frac{L_s}{u_p+ u} $$

**Fig. 3:**
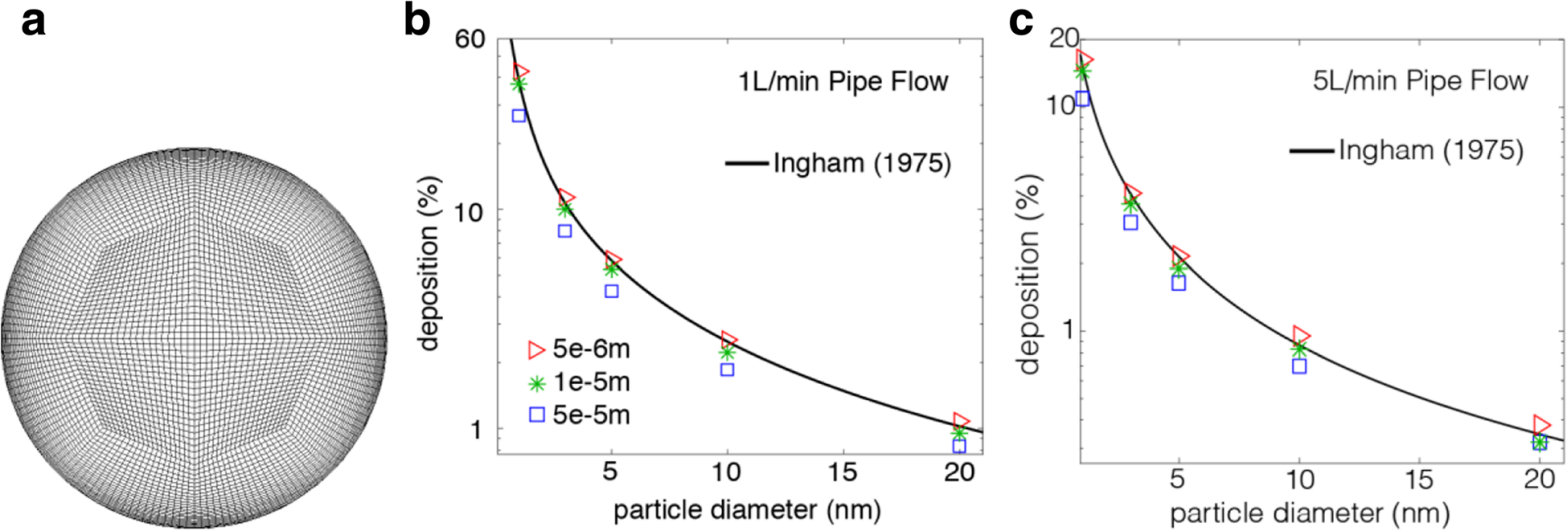
Brownian diffusion validation testing in a pipe geometry. **a** meshing scheme; **b** comparison with analytical solution by Ingham (1975) at pipe flow of 1 L/min; **c** comparison with analytical solution by Ingham (1975) at pipe flow of 5 L/min

This means that the length scale factor is proportional to the integration time step, equivalent to the distance that the particle travels before its equations are solved again and its trajectory updated. A smaller value for the length scale increases the number of calculations per distance length. Its selection must reproduce the diffusion dispersion mechanism for nanoparticles [49]. A standard geometry in the form of a pipe (Fig. 3a) with analytical solution by Ingham (1975) [50] was used to validate the particle dispersion. A fully developed flow of 1 L/min and 5 L/min was used which has a corresponding Re = 312, and Re = 1560 respectively. The particles were introduced into the pipe with a mass flow rate distributed with a fully developed profile as:

7 $$ .\dot{m}(r)={\dot{m}}_0\left(1-\frac{r^2}{R^2}\right) $$

where *m*_0_is the maximum mass flow rate at the pipe centerline, *r* is the radial position from the pipe centerline, and *R* is the pipe radius. Particle deposition in the pipe over a distance of 0.09 m was compared with the deposition efficiency (DE) correlation by Ingham (1975) [50].

8 $$ \mathrm{DE}=1-\left(0.819{\mathrm{e}}^{-14.63\Delta}+0.0976{\mathrm{e}}^{-89.22\Delta}+0.0325{\mathrm{e}}^{-228\Delta}+0.0509{\mathrm{e}}^{-125.9\Delta 2/3}\right) $$

where

9 $$ \Delta =\frac{DL_{\mathrm{pipe}}}{4{U}_{\mathrm{inlet}}{R}^2} $$

Particle deposition in a pipe length of 0.9 m was compared for length scale factors of 5e-5 m, 1e-5 m, and 5e6 m, which showed that the deposition was best described using a value of 1e-5 m. Applying the method to a human pharynx model with 10 different length scale factors, an optimal value of 2e-5 m was identified. Further detail of the methodology was given in [49].

### Particle size distribution and concentration

Typical ambient environment contains polydispersed particles where the number concentration (number of particles per unit volume) is closely related to the size distribution *n(d,* ***r*** *, t)*, given as:

10 $$ dN= n\left( d,\overrightarrow{r}, t\right) d(d) $$

Here *n* is the particle size distribution function, ***r*** is the position, *t* is time and *d* is the particle diameter. Accordingly, total number of particles per unit volume can be obtained as:

11 $$ N={\int}_0^{\infty } n\left( d,\overrightarrow{r}, t\right) d(d) $$

Due to emission, migration and particle coagulation, *N* in ambient environment is a function of time and space. In a time domain from *t*_*1*_ to *t*_*2*_, the averaged size distribution function is given as:

12 $$ \overline{n}\left( d,\overrightarrow{r}\right)=\frac{1}{\left({t}_2-{t}_1\right)}{\int}_{t_1}^{t_2} n\left( d,\overrightarrow{r}, t\right) dt $$

therefore, the total number of particles per unit volume in the timeframe can be obtained as:

13 $$ {N}_{t_1\hbox{-} {t}_2}=\left({t}_2-{t}_1\right){\int}_0^{\infty}\overline{n}\left( d,\overrightarrow{r}\right) d(d)=\left({t}_2-{t}_1\right){\int}_0^{\infty}\left[\frac{1}{\left({t}_2-{t}_1\right)}{\int}_{t_1}^{t_2} n\left( d,\overrightarrow{r}, t\right) d t\right] d(d) $$

Given size distribution function *n(d,* ***r*** *, t)* (*Eq.* *10*), particle surface area, volume and mass concentrations can be readily obtained if particles are spherical, they are:

14 $$ dA=\pi {d}^2\; n\left( d,\overrightarrow{r}, t\right) d(d) $$

15 $$ dV=\frac{\pi}{6}{d}^3\; n\left( d,\overrightarrow{r}, t\right) d(d), $$

16 $$ dM={\rho}_p\frac{\pi}{6}{d}^3\; n\left( d,\overrightarrow{r}, t\right) d(d). $$

Similarly to *Eq. (* *13* *)*, the total surface area, volume and mass of particles per unit volume in the timeframe from *t*_*1*_ to *t*_*2*_ can be obtained as:

17 $$ {A}_{t_1\hbox{-} {t}_2}=\left({t}_2-{t}_1\right){\int}_0^{\infty}\pi {d}^2\overline{n}\left( d,\overrightarrow{r}\right) d(d)=\pi \left({t}_2-{t}_1\right){\int}_0^{\infty}\left[\frac{1}{\left({t}_2-{t}_1\right)}{\int}_{t_1}^{t_2}{d}^2 n\left( d,\overrightarrow{r}, t\right) d t\right] d(d) $$

18 $$ {V}_{t_1\hbox{-} {t}_2}=\left({t}_2-{t}_1\right){\int}_0^{\infty}\frac{\pi}{6}{d}^3\overline{n}\left( d,\overrightarrow{r}\right) d(d)=\frac{\pi}{6}\left({t}_2-{t}_1\right){\int}_0^{\infty}\left[\frac{1}{\left({t}_2-{t}_1\right)}{\int}_{t_1}^{t_2}{d}^3 n\left( d,\overrightarrow{r}, t\right) d t\right] d(d) $$

19 $$ {M}_{t_1\hbox{-} {t}_2}=\left({t}_2-{t}_1\right){\int}_0^{\infty }{\rho}_p\frac{\pi}{6}{d}^3\overline{n}\left( d,\overrightarrow{r}\right) d(d)={\rho}_p\frac{\pi}{6}\left({t}_2-{t}_1\right){\int}_0^{\infty}\left[\frac{1}{\left({t}_2-{t}_1\right)}{\int}_{t_1}^{t_2}{d}^3 n\left( d,\overrightarrow{r}, t\right) d t\right] d(d) $$

### Deposition efficiency and particle dosimetry

Particle deposition efficiency (*DE*) is defined as the ratio of the deposited particles in a region to the total number entering to that region; that is:

20 $$ DE=\frac{Number\  of\  Deposited\  Particles}{Total\  Number\  of\  Particles\  Entering\  to\  the\  Region} $$

It is an important parameter characterizing the regional filtering capacity and particle penetration rate. Deposition efficiency (*DE*) is closely related to the transport mechanisms and for nanoparticles, size, diffusivity and airflow rate are identified the dominant parameters. Due to geometric complexity of human airways, no analytical expression is available for the deposition efficiency (*DE*). Frequently, empirical fitted deposition equations are used to relate measured data (*DE*) to the governing parameters.

Given particle size distribution and airway deposition equation, particle dosimetries by number, surface area, volume and mass can be readily obtained as:

21 $$ {Dose}_{number}=\underset{t_1}{\overset{t_2}{\int }}\underset{d_1}{\overset{d_2}{\int }} n\left( d,\overrightarrow{r}, t\right)(DE) d(d) d t $$

22 $$ {Dose}_{surface\_ area}=\underset{t_1}{\overset{t_2}{\int }}\underset{d_1}{\overset{d_2}{\int }}\pi {d}^2 n\left( d,\overrightarrow{r}, t\right)(DE) d(d) d t $$

23 $$ {Dose}_{volume}=\underset{t_1}{\overset{t_2}{\int }}\underset{d_1}{\overset{d_2}{\int }}\frac{\pi}{6}{d}^3 n\left( d,\overrightarrow{r}, t\right)(DE) d(d) d t $$

24 $$ {Dose}_{mass}=\underset{t_1}{\overset{t_2}{\int }}\underset{d_1}{\overset{d_2}{\int }}{\rho}_p\frac{\pi}{6}{d}^3 n\left( d,\overrightarrow{r}, t\right)(DE) d(d) d t $$

Here (*t*_*1*_ *, t*_*2*_) and (*d*_*1*_ *, d*_*2*_) are the time and particle size range respectively.

## Results

### Particle morphology

**Fig. 4:**
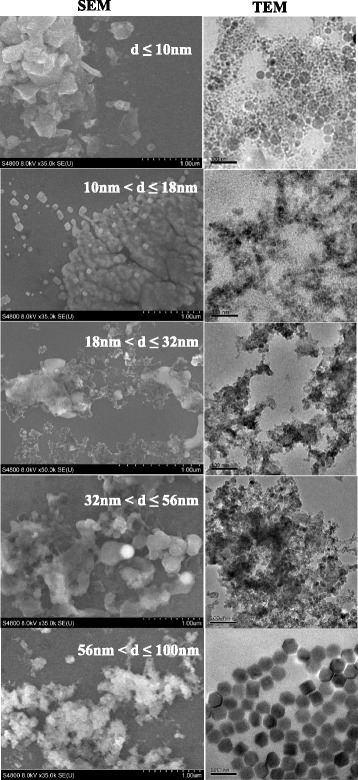
Sample morphologies of collected airborne particles by MOUDI 125B in the WEDM machine shop

Sample SEM and TEM images of the airborne particles were shown in Fig. 4. The aerosol particles from the production activity were largely less than 100 nm and typically captured by the filter of size range from 56 nm to 100 nm. A mixture of iron, aluminum, copper, and trace elements of Mg, Mn, Mo, Zn, Ni, Cr were detected in particle composition. For simplicity, a particle density of 2700 kg/m^3^, close to that of aluminum, was assumed in current numerical simulation. While the larger particles appear to be compact and closer to spherical shapes, the smaller ones are more agglomerate-like formed by clusters of smaller sized spheres. Since the bipolar charger and the particle classification in the SMPS Particle Sizer utilize both a spherical particle model and an idealized aggregated mobility model, both will be considered in the inhalation study. This study focuses on the methodology of the combined study using the spherical assumption. The effect of agglomeration to particle measurement and inhalation risks will be investigated in a subsequent paper.

### Particle distribution in the machine shop

**Fig. 5:**
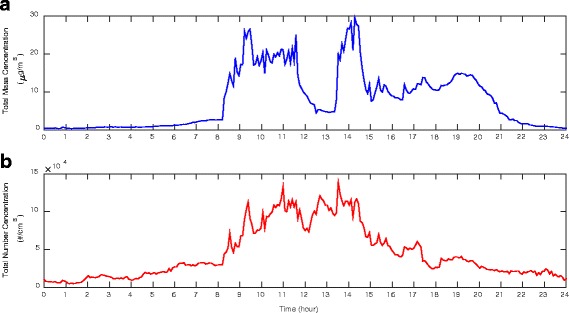
Ultrafine particle (5.52 to 98.2 nm) concentrations during a typical working day: **a** real time ultrafine particle total mass distrubution; **b** real time ultrafine particle total number distribution

Figure 5 shows the measured ultrafine particle (5.52 to 98.2 nm) concentration in the electrical discharge machine shop during a 24-h period in a typical working day. The total mass and number concentrations correlate with working hours which start at 8:00 am and end around 17:30 pm. Particle total mass concentration took a sharp increase (from 2.25 μg/m^3^) shortly after 8:00 am, peaked (at 27 μg/m^3^) around 9:30 am and maintained the high level until lunch break. Total mass concentration decreased steadily during the lunch break and a minimum value of 4.5 μg/m^3^ was reached before 13:00 pm. Particle total mass concentration once again took a sharp increase following the beginning of the afternoon shift, reaching a high of 29.25 μg/m^3^ around 14:00 pm, and dropped to 9 μg/m^3^ around 15:00 pm. The particle concentration was maintained at this level until 20:30 pm before it finally dropped to the background level. A similar trend of variation (from 30,000 to 139,000 particles/cm^3^) was observed in Fig. 5b for the ultrafine particle total numbers; however, a persistent high level of concentration was maintained throughout the working period and it was less affected by the micro-activities such as the lunch break. It was seen from Fig. 5 that both the total mass and total number concentrations for the ultrafine particles in the machine shop correspond to the production activity. A high particle inhalation exposure to the machine operator is clearly demonstrated.

**Fig. 6:**
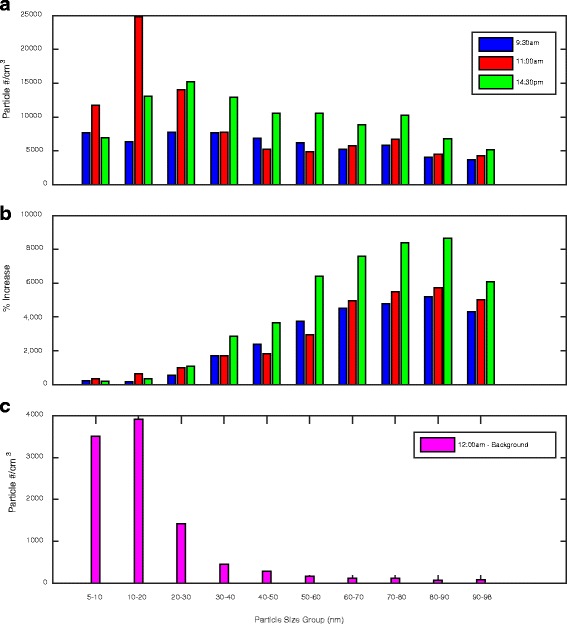
Increase of the ultrafine particle number concentration due to the production activities: **a** particle number increase from the background; **b** percentage of particle number increase from the background

**Fig. 7:**
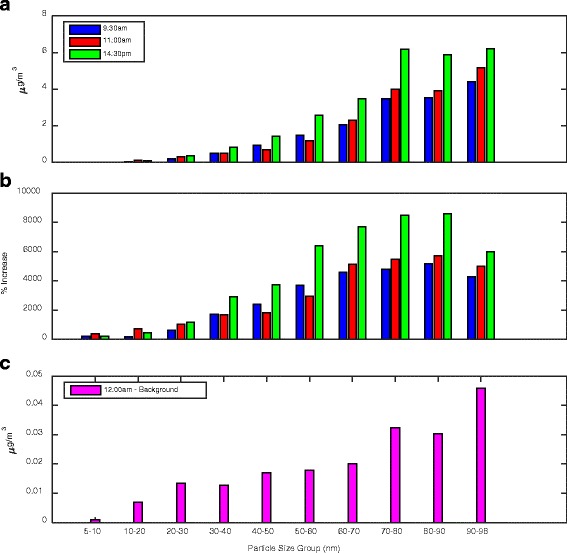
Increase of the ultrafine particle mass concentration due to the production activities: **a** particle mass concentration increase from the background; **b** percentage of particle mass concentration increase from the background

To evaluate the evolution of particle size distribution, Figs. 6 and 7 shows the particle size resolved concentrations at a series of representative high production phases (9:30 am, 11:00 am and 14:30 pm). Background concentration was assumed at midnight when a minimum and steady particle concentration was observed. In these figures, the increase of particle concentration over the background was presented to allow a focused analysis on emissions produced by the machining activity. Figure 6a showed that, across all sized groups, a large number of particles (in the order of 10^4^ #/cm^3^) were generated due to the production. In general, particle number concentration increase was higher in the smaller sized groups (5–30 nm) than the larger ones (>30 nm). However, from the percentage increase perspective (Fig. 6b), the production produced significantly higher number of particles in the larger size groups (>30 nm), monotonically related to the particle sizes. Background particle number concentration was shown in Fig. 6c for comparison. Figure 6 implied a high level of number presence, in the background and also in the machine shop during production, of the ultrafine particles in the lower size range (<30 nm). Relative to the background, the production activity most effectively increased the number of ultrafine particles in the larger size range (>30 nm).

Contrary to the particle number count, increase of the particle mass concentration due to production is clearly positively related to particle size from both absolute and percentage perspectives (Fig. 7). Mass concentration increase for the smaller particle size groups was almost negligible (<20 nm). The mass concentration for the larger particle size groups was monotically increasing with the particle sizes. Figures 6 and 7 implied that though particle number was higher for the smaller sizes, the mass concentration was dominated by the larger sized groups. The mass increase due to production emission was predominantly contributed by ultrafine particles in the larger size range (>50 nm).

### Breathing airflow pattern

**Fig. 8:**
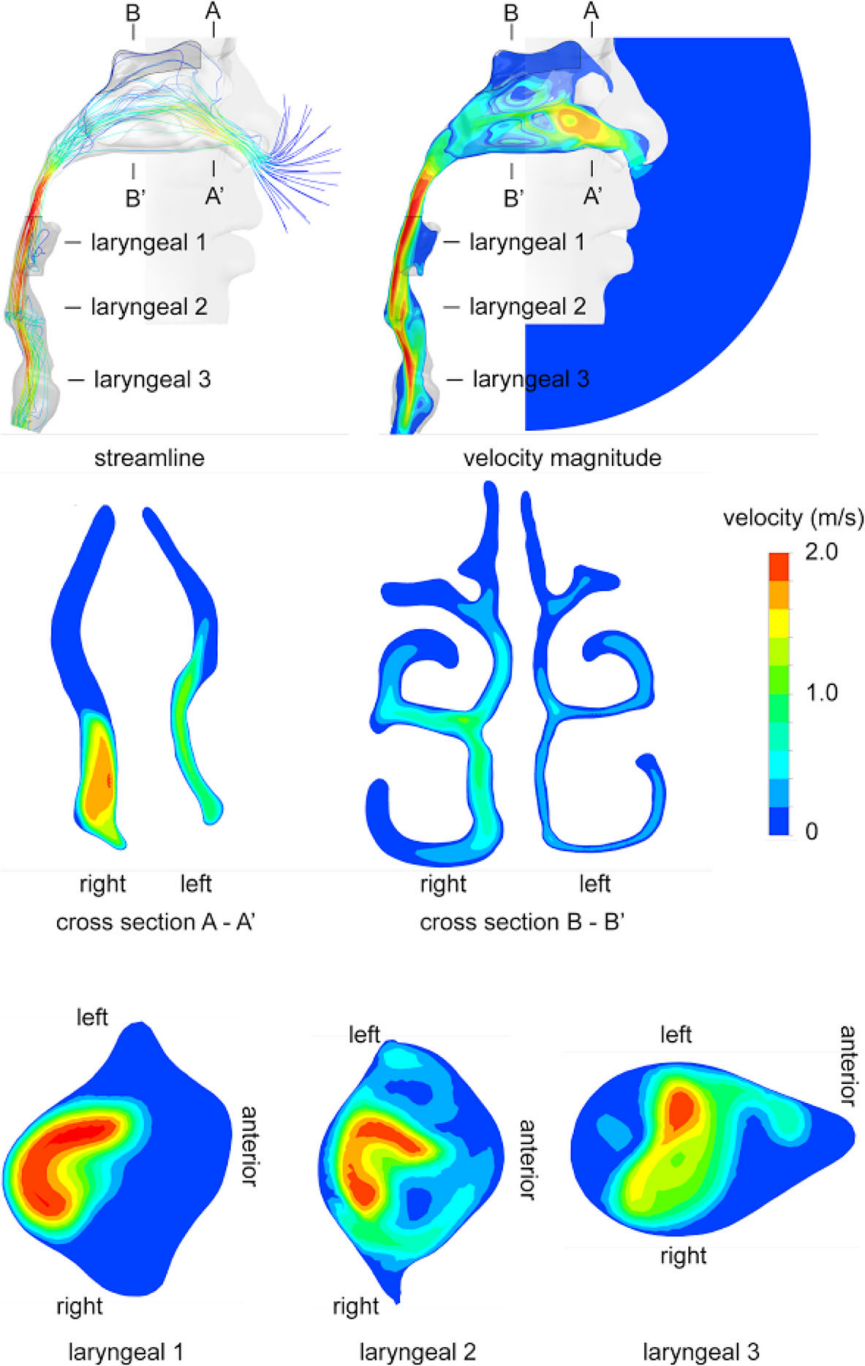
Stream-wise and axial air flow pattern in the nasal and upper airways at selected locations

Light breathing at flow rates of 3 to 12 L/min was included in the simulation. Corresponding Reynolds number at the nostrils was given in Table 1. Key features of the airflow pattern were similar, conforming to the geometric details of the airway. Figure 8 displayed the stream-wise and axial airflow pattern in the nasal and upper airways at selected locations. Ambient air enters the nostril in an upward direction, and turns 90^o^ entering the middle and inferior nasal meatus before a second 90° at the posterior nasopharynx. High velocity was observed at the nostril entrance, downstream of the nasal valve and at the larynx. Bulk air passes through the middle and inferior meatus, while the superior meatus that includes the olfactory region, has very low velocity passing through. Airflow pattern progresses rapidly in the laryngeal region, with high velocity streams shifting from back to anterior walls implying significant secondary flow along the airway passage. Since the inhaled airborne particles are transported by the moving fluid, regions with higher velocity imply high particle concentrations. The flow pattern provides valuable indication for the potential deposition of the inhaled particles. Airflow changes, such as a sharp turn, a sudden contraction or an expansion of the cross sectional area may have profound consequence for particle depositions.

**Table 1:** Airflow rate and Reynolds number

*Airflow rate (L/min)*	*Reynolds number*
3	183
5	305
7.5	458
10	610
12	732

### Particle deposition pattern and deposition equations in human upper airways

**Fig. 9:**
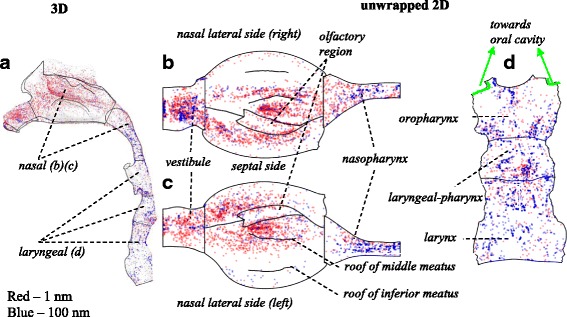
Particle deposition pattern in the nasal and laryngeal region (Q = 10 L/min): **a** nasal and laryngeal region (3D); **b** nasal cavity – left (unwrapped 2D); **c** nasal cavity – right (unwrapped 2D); **d** laryngeal region (unwrapped 2D)

Figure 9 shows sample deposition pattern of the inhaled nanoparticles (1 and 100 nm) in the nasal cavity and laryngeal region. Here particle size range is slightly expanded to allow a wider coverage of the developed deposition equations being applied in future applications. To elucidate the obscured region in the 3D domain (Fig. 9a), a surface mapping technique [51] was applied where the 3D bounding surface is unwrapped to a 2D surface and shown in Fig. 9b-d. High deposition was observed in nasal vestibule, on anterior septum before the 90° turn, and in posterior nasal cavity following the second 90° turn. In main nasal cavity, majority of the deposition occurred in middle meatus. A small fraction was scattered across superior meatus onto the olfactory mucosa. The left and right nasal chamber geometries were asymmetric with the right chamber slightly wider. Particle deposition pattern was affected by particle size with significantly more deposition and random distribution observed for 1 nm particles. Sporadic and streak patterned deposition (in laryngeal region, Fig. 9d) of the 100 nm particles implied a lower level Brownian diffusion. The nasal cavity was shown to effectively filter the 1 nm particles, while 100 nm particles were more likely to penetrate through and have higher deposition in the laryngeal region.

**Fig. 10:**
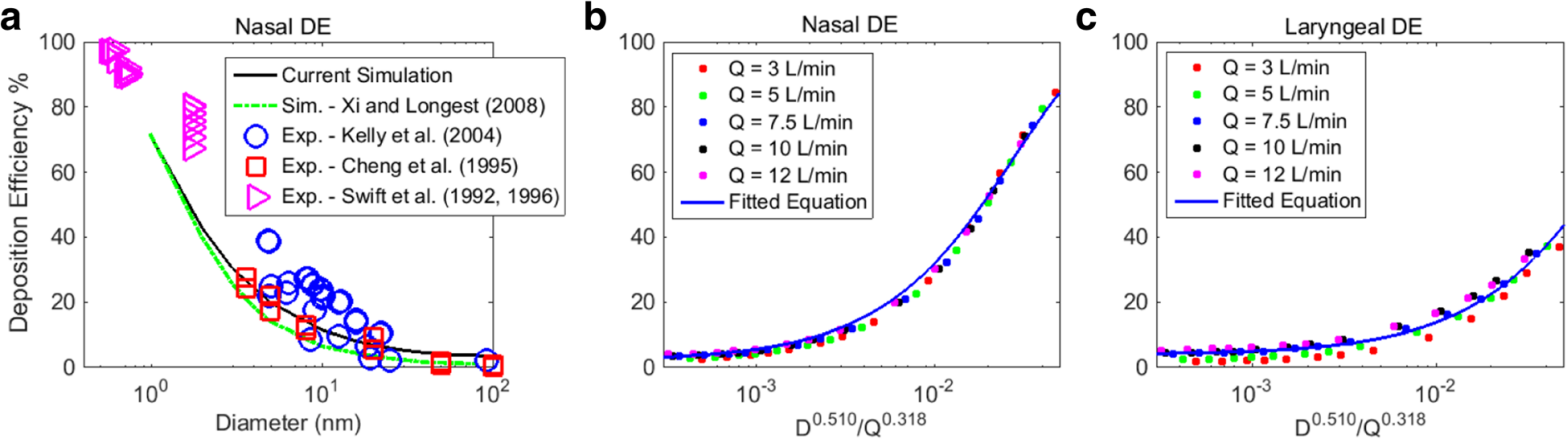
Comparison of nasal deposition efficiencies: **a** nasal deposition efficiencies (Q = 10 L/min); **b** nasal deposition equation; **c** laryngeal deposition equation

Nasal deposition efficiency is defined as the ratio of the deposited particles in nasal cavity to the total number entering through nostril. It is an important parameter characterizing nasal filtering efficiency. Figure 10a presents the current simulation result and the comparison with literature data [35, 52–55]. In the nano range (d < 100 nm), nasal deposition monotonically decreased with increase of particle size and the current simulation agreed well with experimental data. Observed variations, within tolerance of accuracy, were due to the experimental scattering, geometry variation between inhalation subjects, and variation in particle inhalation profiles (far field versus nostril, [56]). Based on the simulation, nasal and laryngeal deposition, as a function of flow rate *Q* (*m* ^*3*^ */s*) and particle diffusivity *D* (*m* ^*2*^ */s*) were developed (Table 2). We find that the correlation *D* ^*0.510*^ */Q* ^*0.318*^ provided the best curve fit for the sampled data for breathing rates of 3 to 12 L/min and particle sizes from 1 to 100 nm in developing the empirical equations (Fig. 10a and b). Similar trends were reported in prior studies, e.g. experiments of Cheng (2003) and simulations from Xi et al. (2008) with the nasal deposition data conforming to *D* ^*0.510*^ */Q* ^*0.280*^ and *D* ^*0.500*^ */Q* ^*0.125*^ respectively [35, 57]. The empirical equations are given as:

**Table 2:** Particle diffusivity D (288.16K)

*Particle Diameter (nm)*	*Diffusivity D (m* ^*2*^ */s)*
1	5.2526E-06
1.5	2.3375E-06
2	1.3165E-06
3	5.8660E-07
5	2.1226E-07
10	5.3757E-08
15	2.4208E-08
20	1.3800E-08
30	6.3013E-09
40	3.6433E-09
50	2.3974E-09
70	1.2935E-09
100	6.8908E-10

25 $$ { D E}_{nasal}=\left(1-0.9793{e}^{-36.51\frac{D^{0.510}}{Q^{0.318}}}\right)\times 100 $$

26 $$ { D E}_{laryngeal}=\left(1-0.9604{\mathrm{e}}^{-10.73\frac{D^{0.510}}{Q^{0.318}}}\right)\times 100 $$

In Eq. (26), the regional laryngeal deposition efficiency is defined as the ratio of the deposited particles in laryngeal region to the total number that entered the region. It is worth to note that Eq. (26) applies to an air flow rate up to 12 L/min. Beyond that, laryngeal induced turbulence start to form which enhances the laryngeal deposition.

### Particle dosimetry in human upper airways in the machine shop

Substitute Eqs. (25) and (26) into Eq. (21), particle number dosimetry in human upper airways can be obtained as:

27 $$ \begin{array}{l}{ D ose}_{nasal\_ numer}=\underset{t_1}{\overset{t_2}{\int }}\underset{d_1}{\overset{d_2}{\int }} n\left( d,\overrightarrow{r}, t\right)\frac{{D E}_{nasal}}{100} d(d) d t\\ {}\kern5em =\underset{t_1}{\overset{t_2}{\int }}\underset{d_1}{\overset{d_2}{\int }} n\left( d,\overrightarrow{r}, t\right)\left(1-0.9793{e}^{-36.51\frac{D^{0.510}}{Q^{0.318}}}\right) d(d) d t\end{array} $$

28 $$ \begin{array}{l}{ D ose}_{laryngeal\_ number}=\underset{t_1}{\overset{t_2}{\int }}\underset{d_1}{\overset{d_2}{\int }} n\left( d,\overrightarrow{r}, t\right)\left(1-\frac{{D E}_{nasal}}{100}\right)\left(\frac{{D E}_{laryngeal}}{100}\right) d(d) d t\\ {}=\underset{t_1}{\overset{t_2}{\int }}\underset{d_1}{\overset{d_2}{\int }} n\left( d,\overrightarrow{r}, t\right)\left(0.9793{e}^{-36.51\frac{D^{0.510}}{Q^{0.318}}}\right)\left(1-0.9604{e}^{-10.73\frac{D^{0.510}}{Q^{0.318}}}\right) d(d) d t\end{array} $$

Similarly, particle dosimetry (by surface area and mass) can be obtained by substituting Eqs. (25) and (26) into Eqs. (22) and (24) accordingly. In the current study, a log-normal particle size distribution (*n)* was detected in the background; however particle size distribution was transient due to machining processes during working hours; therefore, a time averaged particle distribution function based on real time measurement during the 8-h working period was used (Eq. (12)).

**Fig. 11:**
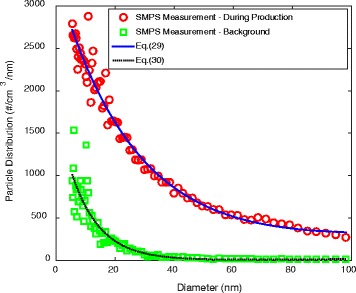
Time averaged particle distribution function in the WEDM machine shop

Figure 11 shows the measured particle distribution by SMPS during production and with the background concentration. Fitted equations shown in Fig. 11 are given as:

29 $$ \begin{array}{l}\overline{n}(d)= a 1\cdot {e}^{b1\cdot d}+ c 1\cdot {e}^{d1\cdot d}\\ {} Here\\ {}\kern2em  a 1= 3197\kern2em  b 1=\hbox{-} 0.03849\kern1.75em  c 1= 130.7\kern1.75em  d 1= 0.006897\end{array} $$

and

30 $$ a 1= 1792\kern1.75em  b 1=\hbox{-} 0.1035\kern2em  c 1= 0.7465\kern1.5em  d 1= 0.03032 $$

Eqs. (29) and (30) give the coefficients for particle size distribution function during production (8-h working period) and at mid-night (background) respectively. Based on the SMPS measurement (Eq. (29)), human upper airway dosimetry in the WEDM machine shop (d = 5.52 - 98.2 nm) was calculated and presented in Table 3. The dosages were based on an 8 h period covering breathing rates of 3 to 12 L/min. Particle penetration, closely related to deep lung dosimetry, was also provided.

**Table 3:** Human upper airway dosages and penetration of nanoparticles from 5.52 to 98.2 nm in the WEDM in a typical working day

*Q (L/min)*	*Number (10* ^*10*^ *#)*			*Mass (*μg*)*			*Surface Area (10* ^*−5*^ *m* ^*2*^ *)*		
	Nasal	Laryngeal	Penetrate	Nasal	Laryngeal	Penetrate	Nasal	Laryngeal	Penetrate
10	3.03	2.04	34.72	1.48	2.87	62.29	6.00	9.99	214.01
12	3.49	2.42	41.84	1.69	3.42	74.85	6.85	11.91	251.24

Table 3 showed a strong monotonic increase in human upper airway dosage (of inhaled nanoparticles) with airflow rate across all metrics. This is simply the result of an increased particle exposure due to larger air exchange. The slight decrease in particle deposition at higher flow rate in diffusion region (Eqs. (25) and (26)) was insignificant to the actual dosimetry. While nasal cavity had a higher “number” dosage than laryngeal, surprisingly, particle mass and surface area dosages in laryngeal were higher than that in the nasal cavity. This difference was overlooked by traditional airway deposition studies where real particle concentration and size distribution were not available. Further examination showed that the slight higher deposition rate of larger sized particles in laryngeal was the cause. For example, at breathing rate of 12 L/min, the deposition efficiencies for 70 nm particles are 3.63% in the nasal and 4.37% in laryngeal region respectively. This implied the mass/surface carrying particles (larger in size) were more likely to pass through the nasal cavity, deposit in high impact region (eg. Laryngeal), or penetrate deep into the lung. It should be noted that empirical fitting could contribute to the increased laryngeal deposition, as a slight under prediction in the nasal and over prediction in the laryngeal region were observed with the fittings (Fig. 10b and c) for low diffusivity particles at low breathing rate (Q = 3 and 5 L/min). Further research is needed in studying the transition region particle deposition, which is extremely low and sensitive to the various transport mechanisms. Here “transition region” refers to the particle size range where the dominant particle transport mechanism changes from Brownian diffusion to inertia, suggested by extremely low particle diffusivity and inertia during the transition phase. More details can be found in the work of Tian and Ahmadi (2007) [58].

**Fig. 12:**
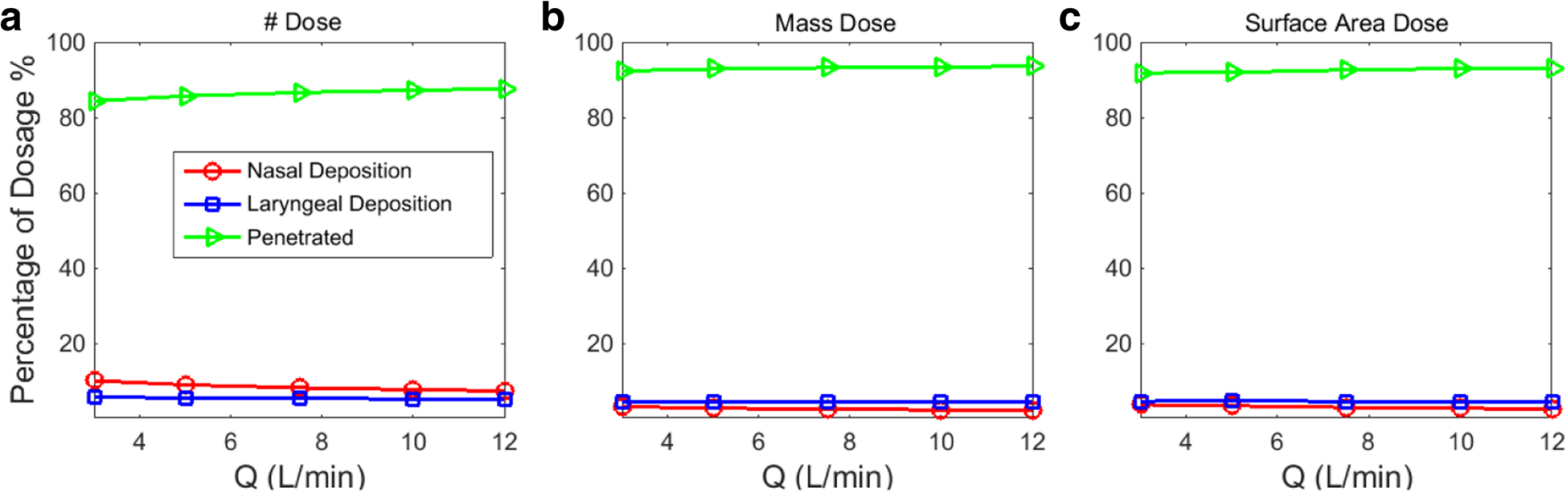
Human upper airway dosage and penetration percentage in the WEDM machine shop: **a** number dosage; **b** mass dosage; **c** surface area dosage

Figure 12 compared the percentage of the dosage (number, mass and surface area) in human upper airway in the WEDM machine shop. Nasal, laryngeal, and the penetration rate (an implication of the deep lung dosage) were considered. Clearly shown in Fig. 12, majority of the particles (d = 5.52 – 98.2 nm) penetrated the nasal and upper airway. Nasal barrier was most effective in reducing particle number intake; however least efficient in trapping mass carrying particles. On the other hand, laryngeal region consistently filtered out number/mass/surface particles in all evaluated metrics. Breathing rate had minimum influence on relative dosimetry. The laryngeal region was the least sensitive to breathing rate, while nasal dosage in particle number count was the most affected by breathing rate.

**Fig. 13:**
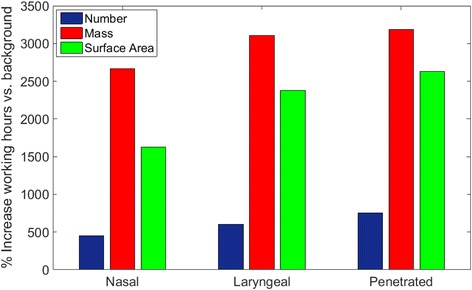
Human airway dosage increase due to production over 8-h shift (Q = 12 L/min)

To examine the effect of production, Table 4 displayed the human upper airway dosimetry with the measured background concentration during 8 h in the working day (d = 5.52 - 98.2 nm). As expected, background dosage estimated from nanoparticle concentration at midnight was significantly lower than the dosage estimated from nanoparticle concentration during working hours (Table 3). To quantify the difference, Fig. 13 presented the percentage increase of the airway doses in the machine shop with respect to the background concentration at a breathing rate of 12 L/min. A remarkable 3100% increase in mass dosage was observed in the laryngeal region, while an even higher percentage increase was seen for the penetrated dose. Meanwhile, a 2664% increase was detected in the nasal cavity. 1626 to 2633% dose increase in surface area, and 451 to 752% dose increase in number was seen respectively. Overall, mass dosage was the most enhanced, and the WEDM production activity had the most profound effects to particle dosage across all regions in all metrics, especially in the laryngeal and downstream airways.

**Table 4:** Human upper airway dosages and penetration of nanoparticles from 5.52 to 98.2 nm with background concentration during 8 h in a day

*Q (L/min)*	*Number (10* ^*10*^ *#)*			*Mass (*μg*)*			*Surface Area (10* ^*−5*^ *m* ^*2*^ *)*		
	Nasal	Laryngeal	Penetrate	Nasal	Laryngeal	Penetrate	Nasal	Laryngeal	Penetrate
10	0.55	0.29	4.07	0.05	0.09	1.89	0.35	0.40	7.83
12	0.63	0.34	4.92	0.06	0.11	2.28	0.40	0.48	9.42

## Discussion

The combined experimental and numerical study showed an astonishing enhancement of human airway dosage as a result of the electrical discharge wire-cutting in the machine shop. At a breathing rate of 12 L/min during a typical 8 h shift, mass dosages to the nasal and laryngeal regions were increased from 0.06 μg to 1.69 μg, and 0.11 μg to 3.42 μg, or 28 and 31 folds respectively. At the same time, mass dosage penetrated deep into the lung was increased from 2.28 μg to 74.85 μg or 33 folds, implying a significant increase of exposure risks to the lower respiratory airways. Though at a relatively milder scale, enhancement of the surface area and number dosages due to production were still significant (6 to 25 folds). Real time particle number and mass concentration increase from the background in the WEDM machine shop (Figs. 6 and 7) has intrinsic effect to airway dosages, which is disproportional to the measured concentration increases when looking from different metric perspectives. This finding implies that a pure number or mass concentration recommendation on the exposure limit at workplace is insufficient. A particle size distribution, together with the deposition equations (eg. Eqs. (25) and (26)), is critical to recognize the actual exposure risks. In addition, human inhalation dosimetry is extremely sensitive to real time particle concentration and size distribution. Averaged particle concentration over 24-h period will inevitably misrepresent the sensible information critical for realistic inhalation risk assessment.

Currently, the most stringent recommendations for ultrafine particle exposure are from National Institute for Occupational Safety and Health (NIOSH, USA) and German Institute for Occupational Safety and Health of the German Social Accident Insurance (IFA). According to NIOSH (2011), the exposure limit for ultrafine titanium dioxide particles (d ≤ 100 nm) is recommended not exceeding 0.3 mg/m^3^ in normal working conditions. IFA (2009) established the benchmark limit for ultrafine particle (1 nm ≤ d ≤ 100 nm, density ≤ 6000 kg/m^3^) concentration at workplace below 40,000 particles/cm^3^. Based on the current measurement, an averaged mass concentration of 0.013881 mg/m^3^ and number concentration of 82,884 particles/cm^3^ were detected in the WEDM machine shop during working hours. Therefore the working condition met the specification of NIOSH; however failed that of the IFA.

As shown in Fig. 13, human airway dosimetry in number, mass and surface area varied significantly. A complete inhalation risk assessment requires the knowledge of toxicity mechanisms in response to each individual metric. For example, a recent study [59] suggested that surface area is the biologically most effective dose metric for nanoparticle lung toxicity. Currently, there is no surface based exposure standard. All recommendations of exposure limits for ultrafine nanoparticles (d ≤ 100 nm) are either on the total mass or number concentrations. Moreover, effects of the particle size distribution and the implication to regional airway dosages, critical for inhalation risk assessment, are not included. Further improvements in these areas are needed.

## Conclusions

The combined experimental and numerical study is the first to establish a realistic exposure condition to calculate the actual particle dosimetry (in mass, number and surface area) by using deposition equations developed through inhalation modeling in a physiologically realistic nasal and upper airway replica. It was found out that particle size distribution carries very important information in determining human airway dosimetry, and critical for inhalation exposure risk assessment. Together with the deposition equations, powerful and accurate prediction of regional dosages with respect to the various dose metrics (e.g. number, mass, and surface area) can be made.

An astonishing enhancement of human airway dosages in the WEDM machine shop was detected by the combined experimental and numerical study. Up to 33 folds in mass, 27 folds in surface area and 8 folds in number doses, penetrating to deeper airways, were detected compared to the background dosimetry. The real time particle concentration measurement showed substantial emission of nano-pollutants by WEDM machining activity, and the combined experimental and numerical study provided extraordinary details on human inhalation dosimetry. It was found out that human inhalation dosimetry is extremely sensitive to real time particle concentration and size distribution. Averaged particle concentration over 24-h period will inevitably misrepresent the sensible information critical for realistic inhalation risk assessment. In the WEDM machine shop, nanoparticle number concentration is dominated by the extremely small scales (d ≤ 30 nm) while mass and surface area concentration is dominated by larger scales (d ≥ 60 nm). Nasal barrier is most effective in reducing particle number intake; however least efficient in catching mass carrying particles. Laryngeal region is consistent in catching particles in all evaluated metrics (number, mass and surface). Majority of the particles (>84% in number, 92% in mass and surface area) (d = 5.52 – 98.2 nm) penetrate into deeper airways. Human upper airway dosages monotonically increase with the breathing rate as a result of the increased particle exposure due to larger air exchange.

Human airway dosimetry in number, mass and surface area varies significantly. A complete inhalation risk assessment requires the knowledge of toxicity mechanisms in response to each individual metric. A pure number or mass concentration recommendation on the exposure limit at workplace is insufficient. A particle size distribution, together with the deposition equations, is critical to recognize the actual exposure risks. For ultrafine nanoparticles (d ≤ 100 nm), all current exposure limit recommendations are either on the total mass or number concentrations, and effects of the particle size distribution and the implication to regional airway dosages, critical for inhalation risk assessment, are not included. Further improvements in these areas are needed.

## Abbreviations

ACGIH: American conference of governmental industrial hygienists
CFD: Computational fluid dynamics
CPC: Condensation particle counter
DMA: Differential mobility analyzers
DPM: Discrete phase model
EC: Electrostatic classifier
EDM: Electrical discharge machining
HEPA: High efficiency particular air filter
IFA: German Institute for Occupational Safety and Health of the German Social Accident Insurance
MOUDI: Micro-orifice uniform-deposit step size impactors
NIOSH: National Institute for Occupational Safety and Health
OSHA: Occupational safety and health administration
SEM: Scanning electron microscopic
SMPS: Scanning mobility particle sizer
TEM: Transmission electron microscopy
WEDM: Wire-cut electrical discharge machine
WHO: World Health Organization

## References

[1] Oberdorster G, Oberdorster E, Oberdorster J (2005). Nanotoxicology: an emerging discipline evolving from studies of ultrafine particles. Environ Health Perspect.

[2] World Health Organization (WHO). Asbestos (Chrysotile, Amosite, Crocidolite, Tremolite, Actinolite, and Anthophyllite). International Agency for Research on Cancer (IARC) Monographs on the Evaluation of Carcinogenic Risks to Humans. 2012;Volume 100c.

[3] Couper J (1837). On the effects of black oxide of manganese when inhaled into the lungs. Br Ann Med Pharmacol.

[4] Wang JD, Huang CC, Hwang YH, Chiang JR, Lin JM, Chen JS (1989). Manganese induced parkinsonism: an outbreak due to an unrepaired ventilation control system in a ferromanganese smelter. Br J Ind Med.

[5] Gorell JM, Johnson CC, Rybicki BA, Peterson EL, Kortsha GX, Brown GG, Richardson RJ (1997). Occupational exposures to metals as risk factors for Parkinson's disease. Neurology.

[6] Meyer-Baron M, Schäper M, Knapp G, Thriel CV (2007). Occupational aluminum exposure: evidence in support of its neurobehavioral impact. Neurotoxicology.

[7] Lucchini RG, Martin CJ, Doney BC (2009). From Manganism to manganese-induced parkinsonism: a conceptual model based on the evolution of exposure. NeuroMolecular Med.

[8] Destaillats H, Maddalena RL, Singer BC, Hodgson AT, McKone TE (2008). Indoor pollutants emitted by office equipment: a review of reported data and information need. Atmos Environ.

[9] Shi XF, Chen R, Huo LL, Zhao L, Bai R, Long DX, Pui DYH, Rang WQ, Chen CY (2015). Evaluation of nanoparticles emitted from printers in a clean chamber, a copy center and office rooms: health risks of indoor air quality. J Nanosci Nanotechnol.

[10] Tönshoff HK, Egger R, Klocke F (1996). Environmental and safety aspects of electrophysical and electrochemical processes. CIRP Ann: Manuf Techn.

[11] Sivapirakasam SP, Mathew J, Surianarayanan M (2011). Constituent analysis of aerosol generated from die sinking electrical discharge machining process. Process Saf Environ Prot.

[12] NIOSH. Current Intelligence Bulletin 63: Occupational Exposure to Titanium Dioxide. Centers for Disease Control and Prevention, National Institute for Occupational Safety and Health. 2011. (http://www.cdc.gov/niosh/docs/2011-160/pdfs/2011-160.pdf).

[13] IFA – Institut für Arbeitsschutz der Deutschen Gesetzlichen Unfallversicherungen. Criteria for assessment of the effectiveness of protective measures; 2009 (http://www.dguv.de/ifa/Fachinfos/Nanopartikel-am-Arbeitsplatz/Beurteilung-von-Schutzma%C3%9Fnahmen/index-2.jsp).

[14] ACGIH (2009). 2009 Tlvs® and Beis® based on the documentation of the threshold limit values for chemical substances and physical agents and biological exposure indices.

[15] OSHA. Metal and metalloid particulates in workplace atmospheres (atomic absorption). Washington, DC: U.S. Department of Labor, Occupational Safety and Health Administration; 2002. (http://www.osha.gov/dts/sltc/methods/inorganic/id121/id121.html).

[16] Heistracher T, Hofmann W (1995). Physiologically realistic models of bronchial airway bifurcations. J Aerosol Sci.

[17] Tian L, Ahmadi G (2012). Transport and deposition of micro-and nano-particles in human tracheobronchial tree by an asymmetric multi-level bifurcation model. J Comput Multiphase Flows.

[18] Zamankhan P, Ahmadi G, Wang Z, Hopke PH, Cheng YS, Su WC, Leonard D (2006). Airflow and deposition of nanoparticles in a human nasal cavity. Aerosol Sci Technol.

[19] Inthavong K, Wen J, Tu JY, Tian ZF (2009). From CT scans to CFD modeling – fluid and heat transfer in a realistic human nasal cavity. Eng Appl Comput Fluid Mech.

[20] Kennedy NJ, Hinds WC (2002). Inhalability of large solid particles. J Aerosol Sci.

[21] Anthony TR, Flynn MR (2006). CFD model for a 3-D inhaling mannequin: verification and validation. Ann Occup Hyg.

[22] Inthavong K, Ge QJ, Li XD, Tu JY (2012). Detailed predictions of particle aspiration affected by respiratory inhalation and airflow. Atmos Environ.

[23] Se CMK, Inthavong K, Tu JY (2010). Inhalability of micron particles through the nose and mouth. Inhal Toxicol.

[24] Inthavong K, Ge QJ, Li A, Tu JY (2013). Source and trajectories of inhaled particles from a surrounding environment and its deposition in the respiratory airway. Inhal Toxicol.

[25] Ghalati PF, Keshavarzian E, Abouali O, Faramarzi A, Tu JY, Shakibafard A (2012). Numerical analysis of micro- and nano-particle deposition in a realistic human upper airway. Comput Biol Med.

[26] Katz IM, Martonen T (1996). Three-dimensional fluid particle trajectories in the human larynx and trachea. J Aerosol Med.

[27] Zhang Z, Kleinstreuer C (2001). Effect of particle inlet distributions on deposition in a triple bifurcation lung airway model. J Aerosol Med.

[28] Hofmann W, Golser R, Balásházy I (2003). Inspiratory deposition efficiency of ultrafine particles in a human airway bifurcation model. Aerosol Sci Technol.

[29] Tian L, Ahmadi G (2013). Fiber transport and deposition in human upper tracheobroncial airways. J Aerosol Sci.

[30] Subramaniam RP, Richardson RB, Morgan KT, Kimbell JS, Guilmette RA (1998). Computational fluid dynamics simulations of inspiratory airflow in the human nose and nasopharynx. Inhal Toxicol.

[31] Matida EA, Dehaan WH, Finlay WH, Lange CF (2003). Simulation of particle deposition in an idealized mouth with different small diameter inlets. Aerosol Sci Technol.

[32] Inthavong K, Tu JY, Heschl C (2011). Micron particle deposition in the nasal cavity using the v2–f model. Comput Fluids.

[33] Ge QJ, Inthavong K, Tu JY (2012). Local deposition fractions of ultrafine particles in a human nasal-sinus cavity CFD model. Inhal Toxicol.

[34] Tian, L, Inthavong, K, Lidén, G, Shang, YD, Tu, JY. Transport and deposition of welding fume agglomerates in a realistic human nasal airway. Ann Occup Hyg. 2016;1–17. (doi:10.1093/annhyg/mew018). 10.1093/annhyg/mew018 27074799

[35] Xi J, Longest PW (2008). Numerical predictions of submicrometer aerosol deposition in the nasal cavity using a novel drift flux approach. Int J Heat Mass Transf.

[36] Inthavong K, Choi L, Ji T, Ding S, Thien F (2010). Micron particle deposition in a tracheobronchial airway model under different breathing conditions. Med Eng Phys.

[37] Häuβermann S, Bailey AG, Bailey MR, Etherington G, Youngman M (2002). The influence of breathing patterns on on particle deposition in a nasal replica cast. J Aerosol Sci.

[38] Tian L, Ahmadi G (2016). Transport and deposition of nano-fibers in human upper tracheobronchial airways. J Aerosol Sci.

[39] Dastan A, Abouali O, Ahmadi G (2013). CFD simulation of total and regional fiber deposition in human nasal cavities. J Aerosol Sci.

[40] Inthavong K, Wen J, Tian Z, Tu JY (2008). Numerical study of fiber deposition in a human nasal cavity. J Aerosol Sci.

[41] Zhong WQ, Yu AB, Liu XJ, Tong ZB, Zhang H (2016). DEM/CFD-DEM modelling of non-spherical particulate systems: theoretical developments and applications. Powder Technol.

[42] Hinds, WC. Aerosol science technology: properties, behavior, and measurement of airborne particles. 2nd ed. Wiley; 1999.

[43] Chen R, Shi XF, Bai R, Rang WQ, Huo LL, Zhao L, Long DX, Pui DYH, Chen CY (2015). Airborne nanoparticle pollution in a wire electrical discharge machining workshop and potential health risks. Aerosol Air Qual Res.

[44] Inthavong K, Tu JY, Ahmadi G (2009). Computational modelling of gas-particle flows with different particle morphology in the human nasal cavity. J Comput Multiphase Flows.

[45] Shang YD, Inthavong K, Tu JY (2015). Detailed micro-particle deposition patterns in the human nasal cavity influenced by the breathing zone. Comput Fluids.

[46] Doorly DJ, Taylor DJ, Schroter RC (2008). Mechanics of airflow in the human nasal airways. Respir Physiol Neurobiol.

[47] Wen J, Inthavong K, Tu JY, Wang S (2008). Numerical simulations for detailed airflow dynamics in human nasal cavity. Respir Physiol Neurobiol.

[48] Li L, Ahmadi G (1992). Dispersion and deposition of spherical particles from point sources in a turbulent channel flow. J Comput Multiphase Flows.

[49] Inthavong K, Tian LP, Tu JY (2016). Lagrangian particle modelling of spherical nanoparticle dispersion and deposition in confined flows. J Aerosol Sci.

[50] Ingham DB (1975). Diffusion of aerosols from a stream flowing through a cylindrical tube. J Aerosol Sci.

[51] Inthavong K, Shang YD, Tu JY (2014). Surface mapping for visualization of wall stresses during inhalation in a human nasal cavity. Respir Physiol Neurobiol.

[52] Kelly JT, Asgharian B, Kimbell JS, Wong B (2004). Particle deposition in human nasal airway replicas manufactured by different methods. Part II: ultrafine particles. Aerosol Sci Technol.

[53] Cheng KH, Cheng YS, Yeh HC, Swift D (1995). Deposition of ultrafine aerosols in the head airways during natural breathing and during simulated breath holding using replicate human upper airway casts. Aerosol Sci Technol.

[54] Swift DL, Montassier N, Hopke PK, Karpen-Hayes K, Cheng YS, Su YF, Yeh HC, Strong JC (1992). Inspiratory deposition of ultrafine particles in human nasal replicate cast. J Aerosol Sci.

[55] Swift DL, Strong JC (1996). Nasal deposition of ultrafine ^218^Po aerosols in human subjects. J Aerosol Sci.

[56] Naseri A, Abouali O, Ghalati PF, Ahmadi G (2014). Numerical investigation of regional particle deposition in the upper airway of a standing male mannequin in calm air surroundings. Comput Biol Med.

[57] Cheng YS (2003). Aerosol deposition in the extrathoracic region. Aerosol Sci Technol.

[58] Tian L, Ahmadi G (2007). Particle deposition in turbulent duct flows – comparisons of different model predictions. J Aerosol Sci.

[59] Schmida O, Stoegera T (2016). Surface area is the biologically most effective dose metric for acute nanoparticle toxicity in the lung. J Aerosol Sci.

